# High protein intake is associated with low prevalence of frailty among old Japanese women: a multicenter cross-sectional study

**DOI:** 10.1186/1475-2891-12-164

**Published:** 2013-12-19

**Authors:** Satomi Kobayashi, Keiko Asakura, Hitomi Suga, Satoshi Sasaki

**Affiliations:** 1 Department of Social and Preventive Epidemiology, Graduate School of Medicine, the University of Tokyo, Tokyo, Japan; 2 Department of Social and Preventive Epidemiology, School of Public Health, the University of Tokyo, 7-3-1 Hongo, Bunkyo-ku, Tokyo 113-0033, Japan; 3 Interfaculty Initiative in Information Studies, the University of Tokyo, Tokyo, Japan

**Keywords:** Protein, Amino acid, Frailty, Brief-type self-administered diet history questionnaire, Elderly Japanese women

## Abstract

**Background:**

Protein intake has been inversely associated with frailty. However, no study has examined the effect of the difference of protein sources (animal or plant) or the amino acid composing the protein on frailty. Therefore, we examined the association of protein and amino acid intakes with frailty among elderly Japanese women.

**Methods:**

A total of 2108 grandmothers or acquaintances of dietetic students aged 65 years and older participated in this cross-sectional multicenter study, which was conducted in 85 dietetic schools in 35 prefectures of Japan. Intakes of total, animal, and plant protein and eight selected amino acids were estimated from a validated brief-type self-administered diet history questionnaire and amino acid composition database. Frailty was defined as the presence of three or more of the following four components: slowness and weakness (two points), exhaustion, low physical activity, and unintentional weight loss.

**Results:**

The number of subjects with frailty was 481 (23%). Adjusted ORs (95% CI) for frailty in the first, second, third, fourth, and fifth quintiles of total protein intake were 1.00 (reference), 1.02 (0.72, 1.45), 0.64 (0.45, 0.93), 0.62 (0.43, 0.90), and 0.66 (0.46, 0.96), respectively (*P* for trend = 0.001). Subjects categorized to the third, fourth, and fifth quintiles of total protein intake (>69.8 g/d) showed significantly lower ORs than those to the first quintile (all *P* <0.03). The intakes of animal and plant protein and all selected amino acids were also inversely associated with frailty (*P* for trend <0.04), with the multivariate adjusted OR in the highest compared to the lowest quintile of 0.73 for animal protein and 0.66 for plant protein, and 0.67-0.74 for amino acids, albeit that the ORs for these dietary variables were less marked than those for total protein.

**Conclusions:**

Total protein intake was significantly inversely associated with frailty in elderly Japanese women. The association of total protein with frailty may be observed regardless of the source of protein and the amino acid composing the protein.

## Background

Frailty is a geriatric syndrome operationally defined by Fried and colleagues as the presence of slowness, weakness, exhaustion, low physical activity, and unintentional weight loss [1]. Because people with frailty have higher risks of subsequent disability, falls, hospitalization, and death than those without frailty [1-3], the prevention of frailty is important in preventing these adverse health outcomes and in meeting the challenge of successful aging in rapidly aging countries, including Japan [4]. Interventions aimed at improving frailty in frail subjects have been conducted [5,6]. Although several exercise programs were shown to improve grip strength and gait speed, which are components of frailty [5], and one exercise and nutritional program improved frailty status over the short (3-months) but not the long term (6- or 12-months) [6], no interventions reported to date have been successful in preventing or improving the state of frailty. The development of more effective strategies for frailty prevention is thus critically dependent on the identification of other factors related to frailty.

A primary cause of frailty might be sarcopenia, the age-related loss of muscle mass and strength [7,8]. A putative cause of sarcopenia is inadequate protein intake [7,8]: a low intake of dietary protein has been associated with a loss of body muscle mass [9], and protein intake has been inversely associated with frailty [10,11]. However, the influence of dietary protein source (animal or plant) and quality (amino acid components) on the prevention of frailty has never been adequately explained. A mixture of essential amino acids appears to consistently enhance muscle protein synthesis and improve physical function. Branched-chain amino acids, including leucine, isoleucine, and valine, are reported to exert beneficial effects on body weight; in particular, leucine plays an important role in muscle protein synthesis. Of the two sulfur amino acids, methionine plays a key role in protein synthesis and cysteine has a role in the synthesis of glutathione, which is essential when specific tissues experience oxidative stress [12]. Impairment of protein synthesis and oxidative stress are thought to cause age-related muscle loss [7], suggesting that these amino acids might be associated with frailty. A better understanding of the association between not only protein but also these individual amino acids to frailty would assist in frailty prevention.

We recently developed an amino acid composition database aimed at estimating the intake of individual amino acids from the Japanese diet [13]. Here, we used this database to evaluate the relationship between protein and amino acid intake and frailty in a large group of elderly Japanese women.

## Methods

### Study population

The present study was based on a cross-sectional multicenter study among three generations, consisting of dietetic students (freshmen), their mothers, and grandmothers. A total of 85 universities, colleges, and technical schools in 35 of 47 prefectures in Japan participated. The survey of institutions in northern and western Japan was conducted from April to May 2011 and that in eastern Japan from April to May 2012. All measurements at each institution were conducted according to the survey protocol. Briefly, a collaborator at each institution explained the general purpose and an outline of the survey to the total of 7016 participants (dietetic students) and distributed a dietary assessment questionnaire and lifestyle questionnaire during the orientation session or a first lecture designed for freshmen in April 2011 or 2012. The collaborators also requested those students able to directly distribute the questionnaires to their mothers and grandmothers to invite their mother and one grandmother to join the study. Recruitment priority was given first to the maternal grandmother; or if unavailable, to the paternal grandmother; or finally to a 65-89 year-old female acquaintance of the student. The student provided written and oral explanations of the general purpose and an outline of the survey to his/her mother and grandmother. Written informed consent was obtained from all participants, and also from a parent for participants aged <20 years. A total of 4933 students, including 4656 women and 277 men (response rate = 70.3%), 4044 women for the mother’s generation (57.6%), and 2332 women for the grandmother’s generation (33.2%) answered both questionnaires. The protocol of the study was approved by the Ethics Committee of the University of Tokyo Faculty of Medicine (No. 3249).

The subjects analyzed in the present study were the participants in the grandmothers’ generation (n = 2332). We excluded those subjects who lived in eastern Japan and answered questionnaires in 2011 (n = 47), because we assumed that they could not report their usual dietary habits and lifestyle due to the occurrence of the Great East Japan Earthquake in March 2011. We also excluded a woman who was in an institution where the response rate was extremely low (2%). Further, we excluded subjects whose age, height, weight, or residential area were missing (n = 20); those aged <65 years (n = 65); and those with a reported energy intake less than half of the energy requirement for the lowest physical activity category according to the Dietary Reference Intakes for Japanese, 2010 (<725 kcal/d; n = 14) [14], or more than 1.5 times of the energy requirement for the highest physical activity category (>3300 kcal/d; n = 32). We further excluded those with Parkinson’s disease (n = 8), chronic kidney disease (n = 13), those who were unable to walk (n = 20), and those with missing information on the variables used for the purpose of multivariate analysis (n = 4). The final sample thus comprised 2108 women aged 65-94 years.

### Dietary assessment

Dietary habits during the preceding month were assessed using a previously validated, brief-type self-administered diet history questionnaire (BDHQ), which can assess the habitual dietary intake [15,16]. Responses to the BDHQ as well as an accompanying lifestyle questionnaire were checked once by research staff at the study office. If any missing or erroneous responses were given to questions which were essential for the analysis, the subject was asked to complete those questions again. Details of the BDHQ’s structure, method of calculating dietary intake, and validity for commonly studied food and nutrient intakes have been published elsewhere [15,16]. Briefly, the BDHQ is a four-page fixed-portion questionnaire used to estimate the dietary intake of 58 food items. To facilitate reading and completion, the present study used a large-print version which increased the size to 10 pages but contained no other changes to structure or content [15,16]. The food items and portion sizes contained in the BDHQ were derived primarily from a food list used in the National Health and Nutrition Survey of Japan and from several recipe books for Japanese dishes [15,16]. Estimates of the intake of the 58 food items and the intakes of energy, total protein, fat, and carbohydrate were calculated using an *ad hoc* computer algorithm for the BDHQ which was based on the Standard Tables of Food Composition in Japan [17]. Protein from fish and shellfish, meat, eggs, and dairy products was included in animal protein. Protein from cereals, pulses, potatoes, confectionaries, fruits, vegetables, alcoholic beverages, and non-alcoholic beverages was included in plant protein. Intakes of eight selected amino acids, namely leucine, isoleucine, valine, methionine, cysteine, branched chain amino acids (sum of leucine, isoleucine, and valine), sulfur amino acids (sum of methionine and cysteine), and essential amino acids were estimated using the answers to the BDHQ and the amino acid composition database [13]. Pearson’s correlation coefficients of protein intake between from the16-d dietary record and from the BDHQ in 92 women aged 31-69 was 0.35 [16], and those of selected amino acids were 0.36 for leucine, 0.34 for isoleucine, 0.34 for valine, 0.31 for methionine, and 0.37 for cysteine (unpublished observations, H. Suga, [13]). Although dietary supplement use was queried in the lifestyle questionnaire, intake from supplements was not included in the analysis due to the lack of a reliable composition table of dietary supplements in Japan. The percentage contribution of each food group to total protein was calculated by dividing daily protein from each food group by daily individual total protein.

### Frailty

Although frailty was operationally defined by Fried and colleagues [1] to include the measures of walking speed for slowness and grip strength for weakness, we did not obtain these measures in our study, but rather used the modified definition developed by Woods and colleagues [2]. Frailty was assessed using the following four components: 1) slowness and weakness (score of the physical functioning scale of the Japanese version short-form 36-item health survey (SF-36) <75 [18-20]); 2) exhaustion (score of the vitality scale of SF-36 <55); 3) low physical activity (those in the lowest quartile); and 4) unintentional weight loss (weight loss in the previous one year >5%). Physical activity was calculated as the average metabolic equivalent-hours, on the basis of the self-reported duration of five activities (walking, bicycling, standing, running, and high-intensity activities) and sleeping and sitting hours over the preceding month, and the metabolic equivalent (MET) value assigned to each activity. These assigned MET values were 3.5 for walking, 7.5 for bicycling, 3.2 for standing, 7.0 for running, 8.0 for high-intensity activities, 1.0 for sleeping, and 1.3 for sitting [21]. Weight loss was calculated from the self-reported weight at the time the BDHQ questionnaire was completed and that one year previously. Subjects with weight loss were asked the question, “Did you lose weight intentionally in the previous year?”, with an answer of no considered to indicate unintentional weight loss. All the questions required to assess frailty except for current weight were incorporated in the lifestyle questionnaires. Current weight was obtained from the response to the BDHQ.

Slowness and weakness was scored as two points, and the other components as one point each. Total frailty score was the sum of all available scores (0-5), with those subjects with a total score ≥3 defined as frail [2].

### Other variables

The subjects reported birth date and body height in the BDHQ. Body mass index (BMI) was calculated as current body weight (kg) divided by the square of body height (m). In the lifestyle questionnaire, the subject reported her residential area, which was grouped into six regions (Hokkaido and Tohoku, Kanto, Hokuriku and Tokai, Kinki, Chugoku and Shikoku, and Kyushu) and also into three categories according to population size (city with a population ≥1 million, city with a population <1 million, and town and village). The subject also reported in the lifestyle questionnaire if she was living alone, as well as her marital status (single, married, widowed, and separated), education (≤junior high school and others, high school, and ≥ college), current smoking status, and dietary supplement use. A history of chronic disease, including stroke, myocardial infarction, hypertension, diabetes, and chronic rheumatism, were considered to be factors which influenced the current state of frailty because the proportions of subjects with histories of these diseases significantly differed between the frail and non-frail group (data not shown). Alcohol drinking was assessed as part of the BDHQ. Depression symptoms were assessed using the Center for Epidemiologic Studies Depression (CES-D) scale [22,23] incorporated in the lifestyle questionnaire, with subjects with a CES-D score ≥16 considered to have depression symptoms.

### Statistical analysis

All nutrient intakes were adjusted for energy by the residual method using a linear regression model [24]. The subjects were divided into quintiles according to each dietary intake. Odds ratios (ORs) and 95% confidence intervals (CIs) for frailty were calculated after adjusting for potential confounding factors. The initial logistic regression model was a crude model into which covariates were added using a forward selection method. The result from a multivariate adjusted model which included a variable of “history of chronic disease (yes or no)”, which indicated the presence of any of the diseases, did not differ to that of a model adjusted for each disease individually as separate variables (data not shown). We therefore treated these diseases as one variable. Final multivariate models were adjusted for age (y, continuous), BMI (kg/m^2^, continuous), residential region (six regions), size of residential area (three areas), living alone (yes or no), current smoking (yes or no), alcohol drinking (yes or no), dietary supplement use (yes or no), history of chronic disease (yes or no), depression symptoms (yes or no), and energy intake (kcal/d, continuous). Survey year (2011 or 2012), marital status (four categories), and education (three categories) were not included in the models, because these variables had no influence on the relationship between dietary variables and frailty (*P* >0.10). All statistical analyses were performed with SAS statistical software, version 9.3 (SAS Institute Inc., Cary, NC, USA). All reported *P* values were two-tailed, with a *P* value of <0.05 considered statistically significant.

## Results

A total of 481 women (22.8%) were classified as frail (Table 1). Mean age (± standard deviation; SD) of the study population was 74.7 (± 5.0) years and mean BMI (± SD) was 22.7 (± 3.2). Mean intakes of protein were 74.0 g/d for total protein, 43.5 g/d for animal protein, and 30.5 g/d for plant protein. Compared with the non-frail group, the frail group was significantly older, had a higher BMI, and included more current smokers and less alcohol drinkers. Further, a higher proportion of subjects with frailty had a history of chronic disease and depression symptoms. The proportion of supplement users was lower among subjects with frailty, and energy intake was lower. Protein and amino acid intakes in the frail group were significantly lower than those in the non-frail group, with respective ratios for each nutrient of 95% - 98% of those in the non-frail group. The main food contributor to total protein in this population was fish and shellfish (30%), cereals (18%), and meat (14%) (Table 2).

**Table 1:** Basic characteristics of 2108 elderly Japanese women categorized with and without frailty

	**Total (n = 2108)**	No frailty* (n = 1627)	Frailty* (n = 481)	***P*** **†**
	**Mean ± SD or n (%)**			
Frailty status*, n (%)				
No frailty	1627 (77.2)	-	-	-
Frailty	481 (22.8)	-	-	-
Frailty criteria, n (%)				
Slowness and weakness	710 (33.7)	234 (14.4)	476 (99.0)	-
Exhaustion	540 (25.6)	205 (12.6)	335 (69.8)	-
Low physical activity	528 (25.1)	228 (14.0)	300 (62.4)	-
Unintentional weight loss	112 (5.3)	50 (3.1)	62 (12.9)	-
Age, years	74.7 ± 5.0	73.9 ± 4.6	77.3 ± 5.3	<0.0001
Body height, cm	150.4 ± 5.5	150.7 ± 5.2	149.1 ± 6.1	<0.0001
Body weight, kg	51.4 ± 7.8	51.4 ± 7.4	51.5 ± 9.2	0.96
Body mass index, kg/m^2^	22.7 ± 3.2	22.6 ± 3.0	23.1 ± 3.6	<0.0001
Survey year, n (%)				0.27
2011	1342 (63.7)	1046 (64.3)	296 (61.5)	
2012	766 (36.3)	581 (35.7)	185 (38.5)	
Residential block, n (%)				0.38
Hokkaido and Tohoku	193 (9.2)	142 (8.7)	51 (10.6)	
Kanto	526 (25.0)	404 (24.8)	122 (25.4)	
Hokuriku and Tokai	510 (24.2)	410 (25.2)	100 (20.8)	
Kinki	262 (12.4)	199 (12.2)	63 (13.1)	
Chugoku and Shikoku	342 (16.2)	265 (16.3)	77 (16.0)	
Kyushu	275 (13.1)	207 (12.7)	68 (14.1)	
Size of residential area, n (%)				0.26
City with a population ≥1 million	274 (13.0)	204 (12.5)	70 (14.6)	
City with a population <1 million	1598 (75.8)	1247 (76.6)	351 (73.0)	
Town and village	236 (11.2)	176 (10.8)	60 (12.5)	
Living alone, n (%)				0.30
No	1759 (83.4)	1365 (83.9)	394 (81.9)	
Yes	349 (16.6)	262 (16.1)	87 (18.1)	
Marital status, n (%)				<0.0001
Single	4 (0.2)	3 (0.2)	1 (0.2)	
Married	1275 (60.5)	1035 (63.6)	240 (49.9)	
Widowed	777 (36.9)	546 (33.6)	231 (48.0)	
Separated	52 (2.5)	43 (2.6)	9 (1.9)	
Education, n (%)				0.04
≤Junior high school and others	977 (46.4)	732 (45.0)	245 (50.9)	
High school	933 (44.3)	733 (45.1)	200 (41.6)	
≥Some college	198 (9.4)	162 (10.0)	36 (7.5)	
Current smoking, n (%)				0.002
No	2054 (97.4)	1595 (98.0)	459 (95.4)	
Yes	54 (2.6)	32 (2.0)	22 (4.6)	
Alcohol drinking, n (%)				<0.0001
No	1695 (80.4)	1278 (78.6)	417 (86.7)	
Yes	413 (19.6)	349 (21.5)	64 (13.3)	
Dietary supplement use, n (%)				0.0002
No	1475 (70.0)	1106 (68.0)	369 (76.7)	
Yes	633 (30.0)	521 (32.0)	112 (23.3)	
Physical activity, total metabolic equivalents-hours/d	39.0 ± 6.6	40.4 ± 6.3	34.4 ± 5.2	<0.0001
History of chronic disease‡, n (%)				<0.0001
No	1059 (50.2)	864 (53.1)	195 (40.5)	
Yes	1049 (49.8)	763 (46.9)	286 (59.5)	
Depression symptoms§, n (%)				<0.0001
No	1617 (76.7)	1363 (83.8)	254 (52.8)	
Yes	491 (23.3)	264 (16.2)	227 (47.2)	
Energy intake, kcal/d	1737 ± 475	1767 ± 473	1636 ± 465	<0.0001
Macronutrient intake||				
Total protein, g/d	74.0 ± 14.3	74.6 ± 14.5	72.0 ± 13.2	0.0003
Animal protein¶, g/d	43.5 ± 15.5	44.0 ± 15.6	41.9 ± 14.9	0.009
Plant protein**, g/d	30.5 ± 4.4	30.6 ± 4.4	30.1 ± 4.6	0.03
Fat, g/d	50.1 ± 9.6	50.3 ± 9.7	49.3 ± 9.2	0.04
Carbohydrate, g/d	241 ± 32	240 ± 32	245 ± 30	0.005
Amino acid intake||				
Essential amino acids, g/d	28.8 ± 6.16	29.0 ± 6.25	28.1 ± 5.77	0.001
Branched chain amino acids, g/d	12.5 ± 2.49	12.6 ± 2.54	12.2 ± 2.32	0.001
Leucine, g/d	5.64 ± 1.12	5.68 ± 1.14	5.50 ± 1.04	0.001
Isoleucine, g/d	3.17 ± 0.65	3.19 ± 0.66	3.08 ± 0.61	0.001
Valine, g/d	3.72 ± 0.72	3.75 ± 0.74	3.63 ± 0.67	0.001
Sulfur amino acids, g/d	2.83 ± 0.55	2.85 ± 0.56	2.77 ± 0.51	0.003
Methionine, g/d	1.76 ± 0.40	1.77 ± 0.40	1.71 ± 0.38	0.01
Cysteine, g/d	1.08 ± 0.15	1.09 ± 0.16	1.07 ± 0.14	0.0007

SD, standard deviation.

*Frailty score (0-5) was defined as the sum of poor physical function (two points), exhaustion (one point), low physical activity (one point), and unintentional weight loss (one point). A score of ≥3 was classified as indicating frailty.

†Means for continuous values were compared by Student’s t test and proportions for categorical values were compared by the chi-square test.

‡History of chronic disease was defined as having had any of the following self-reported diseases: stroke, myocardial infarction, hypertension, diabetes, or chronic rheumatism.

§Depression symptoms were defined as a Center for Epidemiologic Studies Depression score ≥16.

||Nutrient intakes were energy-adjusted according to the residual method.

¶Protein from the following four food groups were included in animal protein: fish and shellfish, meat, eggs, and dairy products.

**Protein from the following eight food groups were included in plant protein: cereals, pulses, potatoes, confectionaries, fruits, vegetables, alcoholic beverages, and non-alcoholic beverages.

**Table 2:** Contribution (%) of each food group to total protein estimated by a brief-type diet history questionnaire among 2108 elderly Japanese women

**Food group**	**Mean ± SD**
Animal food	
Fish and shellfish	30.4 ± 12.1
Meat	14.0 ± 7.15
Dairy products	6.14 ± 4.73
Eggs	6.10 ± 3.72
Plant food	
Cereals	18.1 ± 7.58
Pulses	9.41 ± 4.90
Confectionaries	5.85 ± 4.61
Vegetables	5.57 ± 2.38
Non-alcohol beverages	1.77 ± 1.02
Potatoes	1.52 ± 1.17
Fruits	1.06 ± 0.77
Alcohol beverages	0.07 ± 0.39

SD, standard deviation.

Total protein intake was inversely associated with frailty (Table 3). The multivariate adjusted OR (95% CI) for frailty in the first, second, third, fourth, and fifth quintiles of total protein intake were 1.00 (reference), 1.02 (0.72, 1.45), 0.64 (0.45, 0.93), 0.62 (0.43, 0.90), and 0.66 (0.46, 0.96), respectively (*P* for trend = 0.001). Subjects categorized by the third, fourth, and fifth quintiles of total protein intake (>69.8 g/d) showed significantly lower ORs than those by the first quintile (all *P* ≤0.03). Similarly, animal and plant protein were also inversely associated with frailty (*P* for trend ≤0.04), with the multivariate adjusted OR (95% CI) in the highest compared to the lowest quintile of 0.73 (0.50, 1.06) for animal protein and 0.66 (0.45, 0.95) for plant protein. After further adjustment for animal or plant protein, both protein types remained inversely associated with frailty (*P* for trend ≤0.002), with ORs (95% CI) in the first, second, third, fourth, and fifth quintiles of 1.00 (reference), 1.06 (0.74, 1.51), 0.70 (0.48, 1.01), 0.63 (0.43, 0.92), and 0.61 (0.41, 0.91) for animal protein and 1.00 (reference), 0.64 (0.44, 0.91), 0.50 (0.34, 0.74), 0.60 (0.41, 0.87), and 0.51 (0.34, 0.75) for plant protein. Higher intakes of all selected amino acids were also associated with a lower prevalence of frailty in the multivariate adjusted model (all *P* for trend ≤0.006). The range of adjusted ORs for frailty in the highest compared to the lowest quintile of the amino acids was 0.67 for cysteine to 0.74 for valine. The ORs for these nutrients were less marked than those for total protein.

**Table 3:** Multivariate adjusted odds ratios and 95% confidence intervals for frailty compared to no frailty by quintile of protein and amino acid intakes among 2108 elderly Japanese women

	**Q1 (Lowest) (n = 421)**	**Q2 (n = 422)**	**Q3 (n = 422)**	**Q4 (n = 422)**	**Q5 (Highest) (n = 421)**	***P *** **for trend**
Protein*						
Total protein, g/d	≤62.9	63.0-69.8	69.8-76.1	76.1-84.3	≥84.3	
n of frailty/no frailty†	113/308	117/305	90/332	82/340	79/342	
Age adjusted OR (95% CI)	1.00 (reference)	1.07 (0.78, 1.48)	0.76 (0.54, 1.05)	0.70 (0.50, 0.98)	0.65 (0.46, 0.91)	0.0008
Multivariate adjusted OR (95% CI)‡	1.00 (reference)	1.02 (0.72, 1.45)	0.64 (0.45, 0.93)	0.62 (0.43, 0.90)	0.66 (0.46, 0.96)	0.001
Animal protein§, g/d	≤31.8	31.8-38.8	38.8-45.6	45.6-54.8	≥54.8	
n of frailty/no frailty†	104/317	117/305	94/328	89/333	77/333	
Age adjusted OR (95% CI)	1.00 (reference)	1.20 (0.87, 1.66)	0.88 (0.63, 1.22)	0.86 (0.61, 1.20)	0.71 (0.50, 1.00)	0.008
Multivariate adjusted OR (95% CI)‡	1.00 (reference)	1.12 (0.79, 1.59)	0.76 (0.52, 1.09)	0.71 (0.49, 1.02)	0.73 (0.50, 1.06)	0.009
Plant protein||, g/d	≤27.1	27.1-29.4	29.4-31.2	31.2-33.9	≥33.9	
n of frailty/no frailty†	117/304	102/320	82/340	98/324	82/339	
Age adjusted OR (95% CI)	1.00 (reference)	0.82 (0.59, 1.13)	0.60 (0.43, 0.84)	0.71 (0.51, 0.98)	0.62 (0.44, 0.87)	0.003
Multivariate adjusted OR (95% CI)‡	1.00 (reference)	0.73 (0.52, 1.04)	0.59 (0.41, 0.86)	0.72 (0.51, 1.04)	0.66 (0.45, 0.95)	0.04
Amino acids*						
Essential amino acids, g/d	≤24.1	24.1-27.0	27.0-29.7	29.8-33.3	≥33.3	
n of frailty/no frailty†	108/313	121/301	95/327	80/342	77/344	
Age adjusted OR (95% CI)	1.00 (reference)	1.17 (0.85, 1.61)	0.86 (0.62, 1.20)	0.74 (0.52, 1.04)	0.67 (0.47, 0.94)	0.001
Multivariate adjusted OR (95% CI)‡	1.00 (reference)	1.12 (0.79, 1.59)	0.72 (0.50, 1.05)	0.64 (0.44, 0.93)	0.69 (0.47, 1.00)	0.002
Branched chain amino acids, g/d	≤10.6	10.6-11.8	11.8-12.9	12.9-14.3	≥14.3	
n of frailty/no frailty†	108/313	117/305	96/326	81/341	79/342	
Age adjusted OR (95% CI)	1.00 (reference)	1.12 (0.81, 1.54)	0.85 (0.61, 1.18)	0.74 (0.53, 1.04)	0.68 (0.48, 0.96)	0.003
Multivariate adjusted OR (95% CI)‡	1.00 (reference)	1.06 (0.75, 1.50)	0.75 (0.53, 1.08)	0.63 (0.43, 0.92)	0.70 (0.49, 1.02)	0.004
Leucine, g/d	≤4.78	4.78-5.31	5.31-5.82	5.82-6.44	≥6.45	
n of frailty/no frailty†	110/311	116/306	94/328	82/340	79/342	
Age adjusted OR (95% CI)	1.00 (reference)	1.08 (0.78, 1.48)	0.81 (0.58, 1.23)	0.74 (0.53, 1.04)	0.67 (0.47, 0.94)	0.002
Multivariate adjusted OR (95% CI)‡	1.00 (reference)	1.02 (0.72, 1.45)	0.72 (0.50, 1.04)	0.63 (0.43, 0.92)	0.69 (0.48, 1.00)	0.004
Isoleucine, g/d	≤2.66	2.66-2.97	2.97-3.27	3.27-3.64	≥3.64	
n of frailty/no frailty†	109/312	115/307	95/327	86/336	76/345	
Age adjusted OR (95% CI)	1.00 (reference)	1.09 (0.79, 1.50)	0.83 (0.60, 1.16)	0.80 (0.57, 1.12)	0.64 (0.46, 0.91)	0.002
Multivariate adjusted OR (95% CI)‡	1.00 (reference)	1.07 (0.75, 1.51)	0.74 (0.51, 1.06)	0.69 (0.47, 1.00)	0.68 (0.47, 0.98)	0.004
Valine, g/d	≤3.18	3.18-3.50	3.50-3.83	3.83-4.26	≥4.26	
n of frailty/no frailty†	105/316	121/301	93/329	82/340	80/341	
Age adjusted OR (95% CI)	1.00 (reference)	1.18 (0.86, 1.63)	0.86 (0.62, 1.20)	0.76 (0.54, 1.08)	0.72 (0.51, 1.01)	0.004
Multivariate adjusted OR (95% CI)‡	1.00 (reference)	1.15 (0.81, 1.63)	0.76 (0.53, 1.10)	0.67 (0.46, 0.97)	0.74 (0.51, 1.08)	0.006
Sulfur amino acids, g/d	≤2.41	2.41-2.67	2.67-2.90	2.90-3.23	≥3.24	
n of frailty/no frailty†	102/319	115/307	102/320	81/341	81/340	
Age adjusted OR (95% CI)	1.00 (reference)	1.14 (0.82, 1.58)	0.99 (0.71, 1.38)	0.77 (0.55, 1.08)	0.74 (0.53, 1.05)	0.01
Multivariate adjusted OR (95% CI)‡	1.00 (reference)	1.09 (0.77, 1.55)	0.86 (0.60, 1.23)	0.65 (0.45, 0.95)	0.73 (0.50, 1.06)	0.006
Methionine, g/d	≤1.45	1.45-1.64	1.64-1.81	1.81-2.04	≥2.04	
n of frailty/no frailty†	100/321	119/303	100/322	84/338	78/343	
Age adjusted OR (95% CI)	1.00 (reference)	1.23 (0.89, 1.70)	1.02 (0.73, 1.41)	0.80 (0.57, 1.13)	0.74 (0.52, 1.04)	0.008
Multivariate adjusted OR (95% CI)‡	1.00 (reference)	1.14 (0.80, 1.62)	0.89 (0.62, 1.28)	0.67 (0.46, 0.97)	0.74 (0.51, 1.08)	0.006
Cysteine, g/d	≤0.97	0.97-1.04	1.04-1.11	1.11-1.20	≥1.20	
n of frailty/no frailty†	104/317	120/302	90/332	95/327	72/349	
Age adjusted OR (95% CI)	1.00 (reference)	1.24 (0.90, 1.71)	0.85 (0.61, 1.19)	0.92 (0.66, 1.28)	0.65 (0.46, 0.92)	0.003
Multivariate adjusted OR (95% CI)‡	1.00 (reference)	1.26 (0.89, 1.78)	0.73 (0.50, 1.06)	0.85 (0.59, 1.23)	0.67 (0.46, 0.98)	0.005

CI, confidence interval; OR, odds ratio.

*Protein and amino acid intakes were energy-adjusted according to the residual method.

†Frailty score (0-5) was defined as the sum of poor physical function (two points), exhaustion (one point), low physical activity (one point), and unintentional weight loss (one point). A score ≥3 were classified as frailty.

‡Adjusted for age (y, continuous), body mass index (kg/m^2^, continuous), residential block (Hokkaido and Tohoku, Kanto, Hokuriku and Tokai, Kinki, Chugoku and Shikoku, and Kyushu), size of residential area (city with a population ≥1 million, city with a population <1 million, and town and village), living alone (yes or no), current smoking (yes or no), alcohol drinking (yes or no), dietary supplement use (yes or no), history of chronic disease (any of stroke, myocardial infarction, hypertension, diabetes, or chronic rheumatism; yes or no), depression symptoms (yes or no), and energy intake (kcal/d, continuous).

§Protein from the following four food groups were included in animal protein: fish and shellfish, meat, eggs, and dairy products.

||Protein from the following eight food groups were included in plant protein: cereals, pulses, potatoes, confectionaries, fruits, vegetables, alcoholic beverages, and non-alcoholic beverages.

## Discussion

In this study, 22.8% elderly women were frail. A previous Japanese study [25] which used Fried’s criteria reported a prevalence of frailty among Japanese of 2.7%. However, this study excluded subjects with a dependent (10.0%), and we assume that the actual proportion of frailty in all subjects was actually higher than reported. Other studies [26,27] reported a prevalence of frail subjects of 18.5% and 10.6%, but these used different criteria which were not confirmed to be consistent with Fried’s criteria.

We found that a higher intake of total protein was associated with a lower prevalence of frailty among elderly Japanese women. This association was also observed for both animal and plant protein. Although the intakes of eight selected amino acids were also inversely associated with frailty, the association of total protein was stronger than those of any of these amino acids individually. To our knowledge, this is the first study to examine the association of intake of not only protein but also amino acids with frailty.

To date, three studies have examined the association of protein intake with frailty. Bartali et al. reported that low intake of total protein was significantly associated with frailty in a cross-sectional study [10]. Beasley et al. assessed the effect of protein source (animal protein) or protein quality (essential amino acids) on frailty in a large-scale cohort study, and found that high intake of protein was associated with a lower risk of frailty, and that these associations were independent of animal protein and essential amino acid intake [11]. In contrast, Bollwein et al. showed that amount of protein intake was not associated with frailty, but the distribution (in the morning, at noon, and in the evening) was significantly associated with frailty [28].

Our study showed that the intake of total protein was inversely associated with frailty in a large cohort of elderly Japanese women. Although the intakes of animal and plant protein and individual amino acids were also associated with frailty, these associations were less marked than that of total protein. These results might indicate that the source of protein or kind of amino acid included in dietary protein might not be particularly important in preventing frailty. Many amino acids are assumed to suppress age-related muscle loss by regulating muscle protein synthesis and overcoming oxidative stress [7,12], and the amino acids in the present study might prevent frailty in a cooperative manner. The strong association of total protein with frailty might be due to an additive effect of these amino acids. Meanwhile, previous study showed that vitamin E, vitamin C, and folate were also associated with frailty [10]. Given that many food sources of plant protein are rich in these antioxidants, the effect of plant protein on frailty observed in the present study may have been caused by these antioxidant nutrients included in plant food rather than amino acids.

Japanese government recommends a total protein intake of 50 g/d for women aged ≥70 years [14]. In the present study, subjects who consumed about ≥70 g/d protein had a significantly lower risk of frailty. A previous review study indicated that a protein intake level which meets the nutritional requirements of all healthy individuals does not protect the aged from sarcopenic muscle loss [29]. Although we cannot adequately discuss the appropriate amount of protein intake in this study due to the limited validity of BDHQ, the amount of protein required to prevent frailty might be higher than the recommendation. Distribution of protein intake to meals (in the morning, at noon, and in the evening) may relate to frailty besides the total amount of protein intakes [29]. One recent study supported this hypothesis [28]. Further studies are warranted to clarity both quantitative and qualitative value of protein intake against frailty.

The major strength of our study is that we could examine the relation of protein and amino acid intakes with frailty in a large number of elderly women using multicenter epidemiologic data. Subjects lived over a wide geographical range of Japan and had various dietary and lifestyle habits. Additionally, the dietary questionnaire used has been validated [15,16], albeit that the validity of the intakes of individual amino acids has not been published. Pearson’s correlation coefficients of the amino acids used in the present study were 0.31-0.37 (unpublished observations, H. Suga, [13]), which is comparable to the value for protein (0.35) in the previous validation study [16].

However, several limitations also warrant mention. First, we defined frailty using the score of the physical functioning scale of the SF-36 as a surrogate for walking speed and grip strength, as proposed by Woods and colleagues [2]. Although we were unable to use the most common frailty criteria, developed by Fried and colleagues [1], Woods et al. showed that the physical functioning scale dichotomized at the 25th percentile was strongly associated with poor walking speed and moderately associated with poor grip strength, and maintained that their definition predicted outcomes as well as Fried’s definition [2]. These results may indicate the appropriateness of the criteria we used. Also, the definition of low physical activity was dependent on the distribution of activity level among study subjects. We therefore examined the association of protein and amino acid intake with frailty using different cut-off points for low physical activity. Classification of low physical activity by lowest tertile or lowest quintile produced similar results to those in Table 3 (data not shown). These results suggest that the lowest quartile was an acceptable cut-off point of low physical activity. Second, although we were unable to include the intake of dietary supplements in calculating protein intake, the use of supplements containing mainly protein or amino acid is uncommon in Japanese adult women (1.5%) [30], and any influence of supplements on protein intake may be low. Third, almost all subjects of the present study were grandmothers of selected dietetic students, not a random sample of Japanese elderly women. Not all Japanese adolescents enter college or university (enrollment ratio = 56% [31]) and the grandmothers of students who do might accordingly have a relatively high social and economic status. Thus, our results cannot be readily extrapolated to the general Japanese elderly population. Additionally, the response rate of the grandmothers (33.2%) was low compared to that of the students (70.3%). This might have resulted in a degree of selection bias. The grandmothers who did not participate in the study might have had unhealthy dietary habits and were frail, and this might have attenuated the results. Fourth, although we attempted to adjust for a wide range of potential confounding variables, we were unable to rule out residual confoundings. Finally, the present study was conducted under a cross-sectional design, which prevents the investigation of a causal effect of protein and amino acid intake on frailty. However, we tried to minimize the effect of reverse causality by excluding subjects assumed to be under restricted protein intake (chronic kidney disease) and who had disability (Parkinson’s disease or those who were unable to walk), and also by calculating ORs adjusted for the history of chronic disease.

## Conclusions

We found that total protein intake was inversely associated with frailty in elderly Japanese women. The association of total protein with frailty may be observed regardless of the source of protein and the amino acid composing the protein.

## Abbreviations

BDHQ: Brief-type self-administered diet history questionnaire; BMI: Body mass index; CES-D: Center for Epidemiologic Studies Depression; CI: Confidence interval; MET: Metabolic equivalent; OR: Odds ratio; SD: Standard deviation; SF-36: Short-form 36-item health survey.

## Competing interests

The authors declare that they have no competing interests.

## Authors’ contributions

SK formulated the hypothesis, conducted the study, analyzed and interpreted the data, and wrote the paper. KA contributed to the writing and editing of the paper. HS contributed to the conduct of the research and assisted the analysis. SS was responsible for designing and conducting the study and contributed to the writing and editing of the paper. All authors contributed to the final version of the manuscript. All authors read and approved the final manuscript.
